# Application of deep learning algorithm on whole genome sequencing data uncovers structural variants associated with multiple mental disorders in African American patients

**DOI:** 10.1038/s41380-021-01418-1

**Published:** 2022-01-08

**Authors:** Yichuan Liu, Hui-Qi Qu, Frank D. Mentch, Jingchun Qu, Xiao Chang, Kenny Nguyen, Lifeng Tian, Joseph Glessner, Patrick M. A. Sleiman, Hakon Hakonarson

**Affiliations:** 1 Center for Applied Genomics, Children’s Hospital of Philadelphia, Philadelphia, PA 19104 USA; 2 Department of Pediatrics, The Perelman School of Medicine, University of Pennsylvania, Philadelphia, PA 19104 USA; 3 Division of Human Genetics, Children’s Hospital of Philadelphia, Philadelphia, PA 19104 USA; 4 Division of Pulmonary Medicine, Children’s Hospital of Philadelphia, Philadelphia, PA 19104 USA; 5 Faculty of Medicine, University of Iceland, Reykjavik, Iceland

**Keywords:** ADHD, Depression, Predictive markers, Genetics

## Abstract

Mental disorders present a global health concern, while the diagnosis of mental disorders can be challenging. The diagnosis is even harder for patients who have more than one type of mental disorder, especially for young toddlers who are not able to complete questionnaires or standardized rating scales for diagnosis. In the past decade, multiple genomic association signals have been reported for mental disorders, some of which present attractive drug targets. Concurrently, machine learning algorithms, especially deep learning algorithms, have been successful in the diagnosis and/or labeling of complex diseases, such as attention deficit hyperactivity disorder (ADHD) or cancer. In this study, we focused on eight common mental disorders, including ADHD, depression, anxiety, autism, intellectual disabilities, speech/language disorder, delays in developments, and oppositional defiant disorder in the ethnic minority of African Americans. Blood-derived whole genome sequencing data from 4179 individuals were generated, including 1384 patients with the diagnosis of at least one mental disorder. The burden of genomic variants in coding/non-coding regions was applied as feature vectors in the deep learning algorithm. Our model showed ~65% accuracy in differentiating patients from controls. Ability to label patients with multiple disorders was similarly successful, with a hamming loss score less than 0.3, while exact diagnostic matches are around 10%. Genes in genomic regions with the highest weights showed enrichment of biological pathways involved in immune responses, antigen/nucleic acid binding, chemokine signaling pathway, and G-protein receptor activities. A noticeable fact is that variants in non-coding regions (e.g., ncRNA, intronic, and intergenic) performed equally well as variants in coding regions; however, unlike coding region variants, variants in non-coding regions do not express genomic hotspots whereas they carry much more narrow standard deviations, indicating they probably serve as alternative markers.

## Introduction

Mental disorders are a global health concerns with depression and anxiety disorders costing the global economy of $1 trillion in lost productivity each year [1]. In the United States, serious mental disorders cost the society $193.2 billion each year [2]. Over 13.1 million adults in United States experienced serious mental illness in 2019, and 7.7 million minors (aged 6–17) experienced a mental disorder in 2016 based on statistics from the Centers for Disease Control and Prevention and the National Alliance of Mental illness. Meanwhile, suicide is the second leading cause of death among people aged 10–34 according to the National Institution of Mental Health. Accurate diagnosis is the first and most important step when encountering mental disorders to ensure appropriately tailored therapies; however, the average delay between onset of mental disorder symptoms and treatment is 11 years [3], and the misdiagnosis rate is disappointing [4, 5]. In past few decades, protocols, such as the Diagnostic and Statistical Manual of Mental Disorders (DSM), have improved the mental disorder diagnosis accuracy and efficiency significantly, but unlike many other diseases, objective screening methodologies and lab tests are still lacking for mental disorders due in part to the underlying disease heterogeneity. Also, co-occurrence of different types of mental disorders, e.g., attention deficit hyperactivity disorder (ADHD) and autism [6], make the diagnosis even more challenging. Therefore, alternative diagnostic methods are warranted and could serve as additional reference in the diagnosis of patients with multiple co-occurring types of disorders.

Structural variation in the human genome shows strong association with mental disorders and certain variations have already been leveraged as drug targets [7]. Non-coding structural variants impacting long non-coding RNAs (lncRNAs) have been shown to influence the entire cell cycle by interacting with DNA, RNA, and proteins [8]. The resulting regulatory effects will result in alternation of gene expression in many complex diseases, including but not limited to cancers, Alzheimer’s disease, cardiovascular issues, neuronal disorders, immune responses, and hereditary diseases [9, 10]. Variation and dysregulation in lncRNAs may thus contribute to human complex diseases and may themselves be potential therapeutic targets, e.g., H19, HOTAIR, LUNAR1, MALAT1, NEAT1, MaTARs in cancer [11] and PVT1 in diabetic nephropathy [9]. Mutations in untranslated region (UTR)/intronic regions may also be potential therapeutic targets since they may lead to protein instability [12] or alternative splicing in genes that are critical in signaling pathways, such as tumorigenesis [13]. Meanwhile, machine learning models, especially deep learning algorithms, have been shown to be of potential value in stratifying mental disorders. Researchers have applied machine learning or deep learning algorithms in mental disorders, usually based on one of these four types of feature vectors, i.e., clinical data, genetic/genomic data, vocal and visual expression data, and social media data [14]. Many studies using genetic/genomic data have focused on prioritizing the susceptibility genes and pathways for mental disorders [15, 16]. For studies predicting disease phenotype, the majority are limited to a specific disease type, such as bipolar disorder [17] or ADHD [18]. On the other hand, it is common that a patient may be diagnosed with more than one type of mental disorders, while studies in African American (AA) are also lacking.

In this study, we analyzed blood whole genome sequencing (WGS) data from 4179 ethnic minority individuals (AA), including 1384 patients with the diagnosis of at least one of the eight common mental disorders where we created a multi-layer perceptron (MLP) neuronal network using coding/non-coding structural variation burdens from different genomic regions as feature vectors. This was done to address two questions: first, whether the model could differentiate mental disorder patients and controls; second, whether we could label correctly patients with different types of disorders, especially patients with multiple diagnosis of mental disorders. The accuracy of the prediction was evaluated using two-fold random shuffle tests and our results support a powerful labeling capacity of the deep learning algorithm with non-coding structural variation demonstrating particular robustness to the classification.

## Methods

### Patient cohorts

**Fig. 1:**
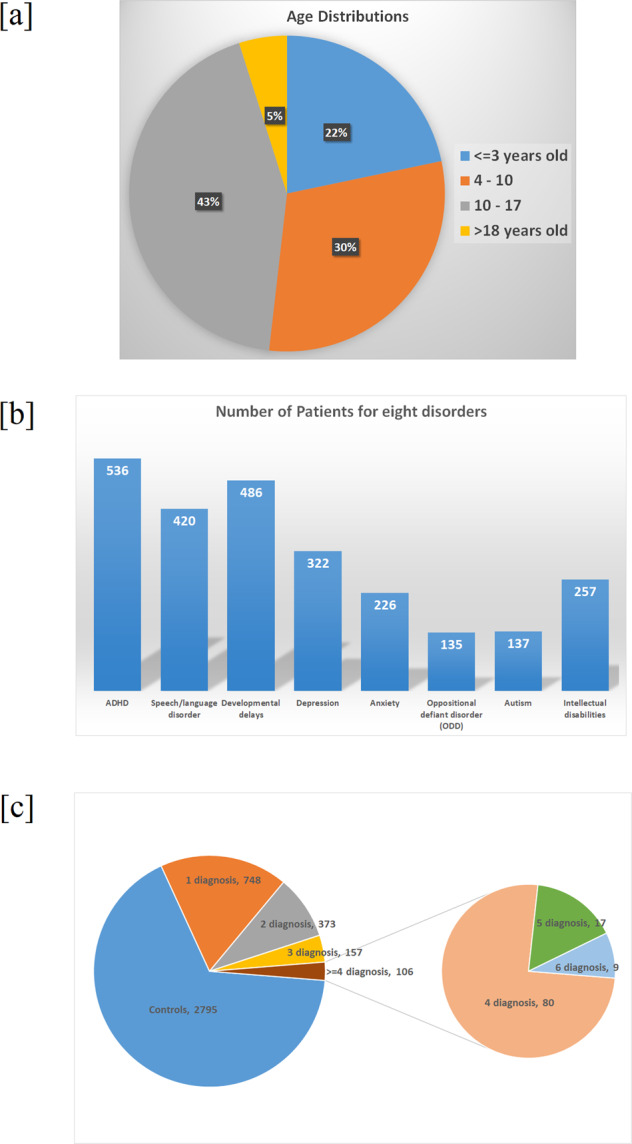
Phenotype summary of 4179 African American individuals from the NHLBI Trans-Omics for Precision Medicine (TOPMed) project. **a** Age distribution of patients: the majority ~95% are children under 18 years old. **b** Number of patients with corresponding eight diagnosis, including ADHD, depression, anxiety, autism, intellectual disabilities, speech/language disorder, delays in developments, and ODD, being noted that one patient could have multiple diagnosis. **c** Distribution of patients’ diagnosis, ranged from controls (no diagnosed mental disorders) to maximum six diagnoses.

The patients selected in this study are from the Center for Applied Genomics (CAG) at The Children’s Hospital of Philadelphia (CHOP), and the WGS was generated through the NHLBI Trans-Omics for Precision Medicine (TOPMed) WGS Program (https://www.ncbi.nlm.nih.gov/projects/gap/cgi-bin/study.cgi?study_id=phs001661.v2.p1). All 4179 AA patients were selected from the CAG biobank, including 1384 patients with a diagnosis of at least one of eight mental disorders (Fig. 1 and Supplementary Table 1). The patients were approached during regular hospital visits at multiple clinics, including emergency room, ambulatory settings, surgical, general pediatrics, and specialty pediatric practices. The patients recruited were in the age range of 0–21 years, obtaining healthcare at CHOP. Parental consent was obtained for individuals under 18 years old and assent was also obtained for subjects aged 7–17 years. The consent allowed samples to be analyzed using the genomic technologies herein, to address the research questions proposed. Parents can opt-in to permit regular updates of their child’s electronic health record data (EHR) and to be re-contacted for future study, which essentially everyone did.

### Electronic health record (EHR) data extractions

The CAG at CHOP maintains a de-identified extract of clinical data from the CHOP EHR database for consented patients. This database contains longitudinal information about visits, diagnoses, medical history, prescriptions, procedures, and lab tests. For this study the mental health status of de-identified individuals was classified based on the International Classification of Diseases (ICD) codes (ICD-9 and ICD-10) associated with clinical visits and entered in the medical history record.

### Whole genome sequencing (WGS) data processing and variation detection

The WGS variant call format files were extracted from the TOPMED database directly. Based on the description, the DNA was isolated from whole blood, and DNA quantity and sex discordance have been checked in the quality assessments. Libraries for WGS were created using the Illumina’s TruSeq DNA PCR-Free Library Preparation Kit. WGS was performed on the Illumina HiSeq X Ten platform with paired end 150 bp reads. The bcl2fastq v2 15.0 package was used to generate individual FASTQ files. The alignment pipeline can be found at https://github.com/CCDG/Pipeline-Standardization/blob/master/PipelineStandard.md. The common variants that have minor allele frequency greater than 0.05 in AA ethnicity based on the Exome Aggregation Consortium database [19] have been removed.

### Genomics feature vectors selections for deep learning models

The human genome was divided into 587 pieces (~5M bp/piece) based on the GRCh38 genomic coordinates. The occurrence classified seven different types of variation, including nonsynonymous single nucleotide variants (SNV), frameshift SNV, SNVs in UTR, non-coding RNA SNV, SNV in intronic region, SNV in intergenic region, and SNV producing a stop codon, for each genomic piece. The genomic pieces were subsequently applied as a feature vector in the deep learning model. The processes were repeated for all individuals in the study. A random forest algorithm was applied to reduce the number of feature vectors by computing relative importance or contribution of each genomic piece in the prediction, then we scaled the relevance down so that the sum of all scores is 1. Feature vectors with zero relative importance were removed for different types of variants. Technically, the random forest model uses “gini” to measure the quality of a split, while the minimum number of samples required to split an internal node equals 2, and nodes are expanded until all leaves are pure or until all leaves contain less than 2. The number of features to consider when looking for the best split equals the square root(num_features) and the number of trees in the forest equals 500. The modeling codes are based on the Scikit-learn package (version 0.21.3, https://scikit-learn.org/) in Python language. Feature vectors with the highest weights were considered as hotspots, and drug target genes within the hotspot regions were explored through the Integration of the Drug–Gene Interaction Database [20]. Only FDA-approved medications were considered.

### Deep learning parameters and random shuffled two-fold tests

MLP from the Scikit-learn package (version 0.21.3) was applied as the deep learning model based on seven different types of variants. Two types of prediction have been made including binary labeling of patients diagnosed with mental disorders versus controls, and multiple labeling for patients with at least one type of mental disorders, including ADHD, depression, anxiety, autism, intellectual disabilities, speech/language disorder, delays in developments, and oppositional defiant disorder (ODD). Thus, each of 1384 patient’s phenotype becomes a 1 × 8 binary matrix instead of a binary value and each column corresponds to one of eight disorders as described above. Parameters for the deep learning model, including maximum iterations, alpha value in L2 regularization, activation functions, solvers, learning rate, number of layers, and numbers of neurons per layer, were optimized using “gp_minimize” function from the scikit-optimize 0.7.2 python library.

In order to test the predictive abilities for selected features, we applied a two-fold shuffle testing. More specifically, 1384 patients and 2795 controls were split into 1:1 ratio for 50 rounds randomly for case–control labeling, with one set used as training data and the other one used as independent test set. The genomic feature vectors were selected as described in the previous paragraph for training data, then the deep learning model described above is applied to label whether the sample is a mental disorder patient or control in the testing data. Similarly, for multiple labeling of 1384 patients with at least one diagnosis, these samples were split into 1:1 ratio for 50 rounds randomly, instead of generating a binary value labeling, the prediction output is a 1 × 8 matrix, while each column corresponds to one of the eight disorders, and value 1 represents existence of the disorder.

## Results

### Phenotype prediction accuracy for mental disorders versus controls in 4179 African American (AA) individuals using two-fold shuffle tests

As described in the Method section, two-fold shuffle testing was applied to assess the mental disorders’ prediction, based on 50 rounds of two-fold random shuffle tests of genetic variants. Reduced feature vectors, which were based on the random forest algorithm, showed a reproducible prediction accuracy at 65% in classifying mental disorder patients versus controls using the deep learning model (Table 1) with optimized parameters as described in the Method section. A notable observation is that structural variants in non-coding regions, such as variants in non-coding RNAs, intronic and intergenic regions, showed similar level of predictive accuracy compared to structural variants in coding regions, including nonsynonymous SNV, frameshift SNVs, and SNVs producing stop codons.

**Table 1:** Prediction accuracy summary (mean ± standard deviation).

Variation types	Prediction accuracy (single diagnosis mental disorder vs controls)	Prediction accuracy (mental disorder vs controls)	Prediction accuracy (hamming loss among 8 disorders)	Prediction accuracy (exactly matches among 8 disorders)
Nonsynonymous SNVs	71.7 ± 1.34%	64.5 ± 1.2%	0.28 ± 0.011	8.8 ± 1.1%
Frameshift SNVs	70.8 ± 1.61%	64 ± 1.4%	0.30 ± 0.007	8.4 ± 0.91%
Stop codon SNV	71.49 ± 1.69%	65.1 ± 0.97%	0.28 ± 0.004	7.2 ± 0.69%
SNVs in UTR region	72.4 ± 1.44%	65.5 ± 1.1%	0.31 ± 0.009	7.6 ± 1.2%
SNVs in ncRNA	72.6 ± 1.52%	65.7 ± 1.3%	0.29 ± 0.009	8.1 ± 1.4%
SNVs in intronic regions	72.8 ± 1.29%	65.7 ± 1.1%	0.28 ± 0.008	8.1 ± 0.98%
SNVs in intergenic regions	73.1 ± 1.23%	64.6 ± 1.1%	0.30 ± 0.006	9.3 ± 0.90%

### Phenotype prediction accuracy for patients with multiple diagnosis in 1384 African American (AA) individuals using two-fold shuffle tests

Unlike labeling of patients versus controls, which is a binary question, labeling patients with multiple diagnosis is a multi-labeling question. More specifically, instead of having a binary value representing presence/absence of the disorders, the phenotype of each patient is a 1 × 8 binary matrix, with each column corresponding to one type of disorders in the order of ADHD, speech/language disorders, developmental delays, depression, anxiety, ODD, autism, and intellectual disabilities. As a result, the accuracy of prediction is more complicated to present. We applied hamming loss, which is considered a standard accuracy representative that is frequently applied for binary multiple labeling question to measure the prediction accuracy. The hamming loss is the fraction of labels that are incorrectly predicted, which is ranged from 0 to 1, while lesser value of hamming loss indicates a better classifier. As shown in Table 1, the hamming loss score is less than 0.3, indicating high fractions of correct labeling. Meanwhile, we also calculated the exact matches of phenotype labeling, to determine if a patient diagnosed for ADHD, autism, and ODD, has a predictive phenotype that is exactly the same as the diagnosis. The accuracy ranged from 7 to 10% depending on the variant types. Considering random guess accuracy for the phenotype is 1/256 (~0.4%), the deep learning model has superior prediction capacity compared to random guesses. The accuracy and the recall for eight different disorders are shown in Tables 2 and 3 for coding and non-coding variants, respectively.

**Table 2:** Prediction accuracy for specific disorders in patients with at least one diagnosis based on coding variants.

	Nonsynonymous SNVs		Frameshift SNVs		Stop codon	
Disorder	Precision	Recall	Precision	Recall	Precision	Recall
ADHD	39.4 ± 2.7%	40.5 ± 5.1%	39.8 ± 2.8%	39.1 ± 3.3%	31.3 ± 40.2%	0.08 ± 0.27%
Speech/language disorders	30.3 ± 3.4%	30.6 ± 5.2%	32.6 ± 3.1%	30.9 ± 4.2%	0.0 ± 0.0%	0.0 ± 0.0%
Developmental delays	36.8 ± 2.1%	37.1 ± 4.2%	36.4 ± 2.8%	36.2 ± 4.3%	33.6 ± 1.2%	90.1 ± 1.9%
Depression	25.9 ± 3.1%	23.6 ± 4.4%	26.3 ± 3.4%	24.8 ± 3.6%	19.9 ± 6.5%	3.7 ± 1.5%
Anxiety	18.1 ± 4.1%	13.7 ± 6.1%	20.6 ± 3.4%	18.9 ± 4.1%	15.1 ± 2.3%	14.9 ± 2.9%
ODD	13 ± 11.1%	6.1 ± 5.1%	20.6 ± 5.9%	14.6 ± 4.7%	17.4 ± 9.7%	2.5 ± 1.5%
Autism	11.9 ± 7.1%	6.1 ± 4.8%	15.8 ± 4.5%	13.2 ± 4.6%	11.2 ± 2.6%	12.2 ± 3.4%
Intellectual disabilities	20.3 ± 3.8%	19.4 ± 5.3%	21.4 ± 2.9%	18.8 ± 4.4%	0.0 ± 0.0%	0.0 ± 0.0%

**Table 3:** Prediction accuracy for specific disorders in patients with at least one diagnosis based on non-coding variants.

	SNVs in UTR region		SNVs in ncRNA		Intronic SNVs		Intergenic SNVs	
Disorder	Precision	Recall	Precision	Recall	Precision	Recall	Precision	Recall
ADHD	38.4 ± 2.5%	33.4 ± 7.3%	39.6 ± 2.7%	32.4 ± 5.7%	39.3 ± 3.6%	31.1 ± 7.1%	42.6 ± 2.3%	42.8 ± 3.9%
Speech/language disorders	30.8 ± 3.3%	31.6 ± 5.8%	30.1 ± 2.9%	26.5 ± 5.3%	30.6 ± 2.8%	31.8 ± 5.4%	33.5 ± 2.8%	33.6 ± 4.5%
Developmental delays	35.4 ± 3.5%	33.5 ± 7.3%	35.4 ± 2.5%	38.4 ± 4.5%	36.6 ± 3.1%	35.5 ± 4.5%	36.4 ± 2.4%	36.8 ± 4.2%
Depression	24.1 ± 2.9%	25.9 ± 7.6%	24.6 ± 2.8%	26.9 ± 4.7%	24.7 ± 3.9%	24.7 ± 5.5%	26 ± 3.1%	26.7 ± 5.0%
Anxiety	18.6 ± 3.7%	17.2 ± 4.2%	17.2 ± 6.6%	6.8 ± 3.9%	16.1 ± 12.3%	3.8 ± 3.2%	20.1 ± 4.4%	17.9 ± 4.4%
ODD	10.9 ± 5.5%	10.3 ± 6.2%	11.6 ± 4.8%	9.6 ± 5.5%	6.8 ± 9.1%	3.2 ± 2.5%	15.4 ± 4.4%	13.5 ± 4.1%
Autism	12.9 ± 4.9%	10.6 ± 5.2%	13.2 ± 4.8%	9.5 ± 3.6%	14.9 ± 7.2%	7.9 ± 4.2%	16.9 ± 5.3%	13.6 ± 4.3%
Intellectual disabilities	19.9 ± 3.4%	14.8 ± 5.3%	22.1 ± 7.9%	10.5 ± 5.3%	21.8 ± 5.8%	9.8 ± 3.5%	20.5 ± 2.9%	22.2 ± 4.4%

### Genomics regions with high weights based on the deep learning model

**Fig. 2:**
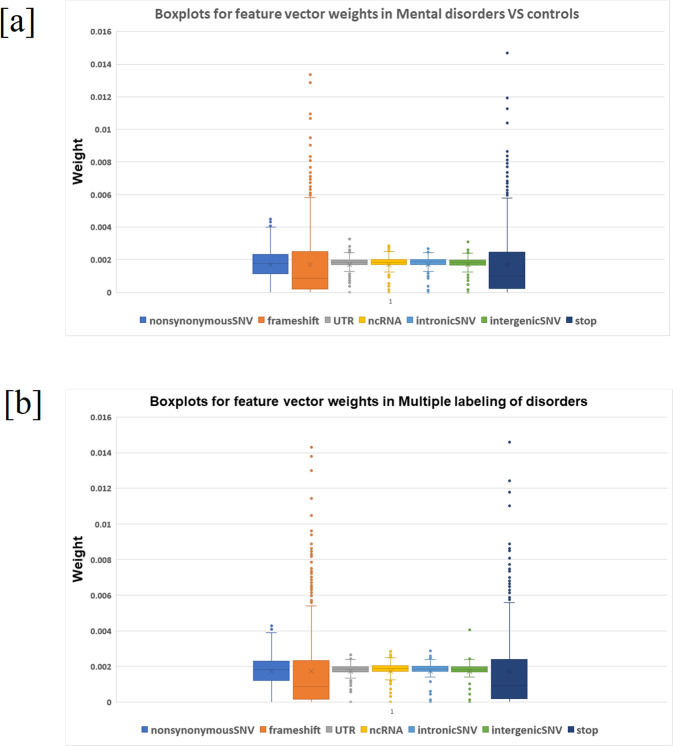
Boxplots for weights of 587 genomic regions (feature vectors). **a** In prediction of cases versus controls. **b** In multiple labeling for 1384 mental disorder patients.

**Fig. 3:**
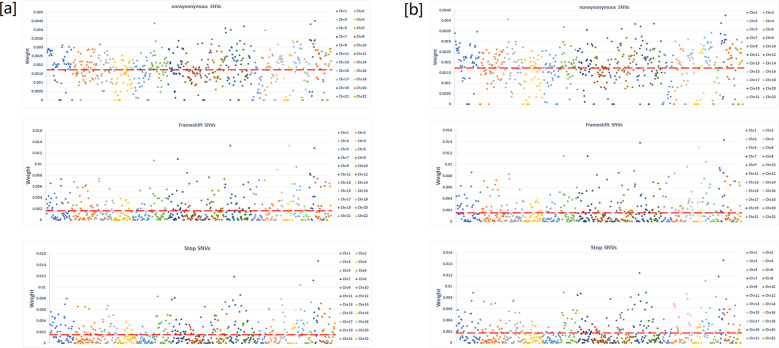
Feature vector weight distribution of three different types of structural variants (nonsynonymous SNVs, frameshift SNVs, and stop codon SNVs) cross 22 autosomes. **a** In prediction of cases versus controls, **b** In multiple labeling for 1384 mental disorder patients. Red dash line is the value if the genomic regions are uniformly weighted.

**Fig. 4:**
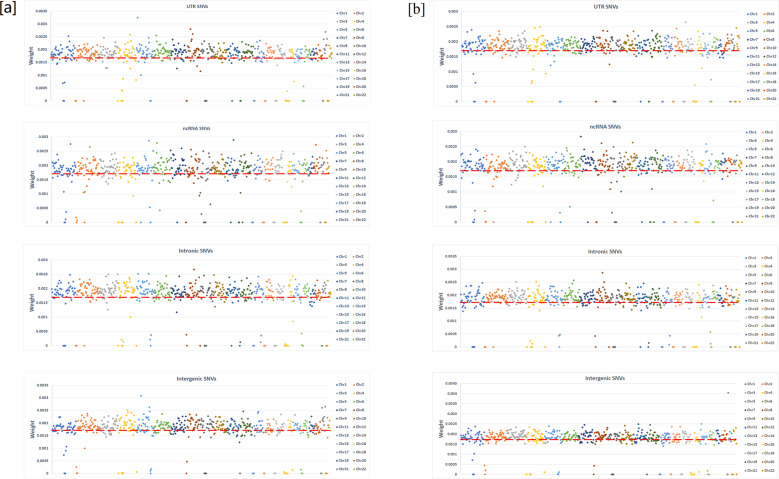
Feature vector weight distribution of four different types of structural variants (SNVs in UTR regions, ncRNA, intronic regions, and intergenic regions) cross 22 autosomes. **a** In prediction of cases versus controls. **b** In multiple labeling for 1384 mental disorder patients. Red dash line is the value if the genomic regions are uniformly weighted.

The weight or the contribution of each genomic region (feature vector) is based on the 4179 AA individuals and calculated using the Random Forest algorithm, as described in the Method section. The genomics regions (as feature vectors) containing variants that showed non-uniformed weights in both prediction models (case–control and multiple labeling) and the weights of variants in coding regions have larger standard deviations than that of variants in non-coding regions. In other words, genomic regions with non-coding variants (UTR/ncRNA/intronic/intergenic) show more uniformed weight distribution compared to regions with coding region variants (Fig. 2). This suggests that variants in non-coding regions mainly serve as biomarkers of genetic susceptibility of mental disorders, conferred by functional genetic variants in each region. In addition, different chromosomes show alternative patterns of weights, and a notable fact is that the coding hotspots were almost same between case–control classification and multiple labeling models (Fig. 3). This is in contrast to the patterns of hotspots that are not matched for non-coding variants between the two models (Fig. 4). Enrichment analysis was performed based on gene hotspots (>1% weight) using the DAVID Bioinformatics platform [21]. Training the models in computer clusters will only take a few hours (less than 1 day on a standard PC). The computational time includes mainly feature vector extractions and parameter optimizations. In the feature vector extraction step, the programs must scan through the WGS data to annotate and categorize the SNVs, therefore consuming a huge amount of computational time and resources (about 5 days on clusters). Parameter optimization using the “gp_minimize” function from the scikit-optimize 0.7.2 python library takes about 3 days since many parameters, especially number of neuros and layers, need to be tested.

## Discussion

Accurate diagnosis of mental disorders can be difficult, and even more challenging in patients who suffer comorbid conditions with more than one type of mental disorders. Although guidelines and standards based on the DSM are helping, the misdiagnosis rate is still high. An assessment of 840 patients in 2011 showed that the misdiagnosis rates reached 65.9% for major depressive disorder, 92.7% for bipolar disorder, 85.8% for panic disorder, 71.0% for generalized anxiety disorder, and 97.8% for social anxiety disorder [5]. A more recent study showed that 51% of schizophrenia had primary diagnosis in the consultation clinic different from the following visits [22], and the misdiagnosis of ADHD is also high, including both over and under estimations [23]. The misdiagnosis could result in prescription of wrong medications that can lead to side effects from the medication without any of the benefits, then further worsen the condition as a consequence [24]. The difficulties in diagnosing mental disorders are further complicated by comorbid symptoms heterogeneity, and lack of objective standards like imaging/lab testing methodologies that are commonly useful for other diseases. For young patients, especially toddlers under 3 years of age who are not able to finish any writing tests for mental disorders, the delay and misdiagnosis rates are even more serious. This is unfortunate as early intervention is critical for many types of severe mental disorders. For example, a previous study shows that early intervention before 30 months of age could significantly improve IQ, an adaptive behavior in autism [25]. As a result, objective alternative approaches could serve as independent references to aid the clinicians to reduce the misdiagnosis rate and make correct decisions for young patients and toddlers. Over the past 15–20 years, structural variants in the genome, including both coding/non-coding regions, have been identified and used as biomarkers in informing the diagnosis and treatment course of mental disorders [26, 27]. In this study we combed genomic variants identified from 4179 AA, with 22% of patients under age 3 years (Fig. 1a) and applied as feature vectors in two MLP deep learning models, which label mental disorder patients versus controls, and patients with multiple mental disorders, respectively.

Among the 4179 AA individuals, we selected 1384 patients who were diagnosed with at least one of eight common mental disorders: ADHD, depression, anxiety, autism, intellectual disabilities, speech/language disorder, delays in developments, and ODD (Fig. 1b). In the first prediction model of mental disorders versus controls, the prediction model showed average accuracy around 65% based on 50 rounds of two-fold random shuffle tests for variants in coding and non-coding regions (Table 1). The accuracy is lower than the previous study labeling of ADHD versus control (~80%) [18]. The main reason is likely due to the comorbid factors when combining eight disorders together as cases that cause significant increase in genetic heterogeneity.

The second prediction model clarified a more interesting question, which is whether we could predict the diagnosis for patients with multiple disorders. In other words, a single patient could belong to multiple categories. Hamming loss, which is the fraction of labels that are incorrectly predicted and frequently applied as accuracy standards for multiple labeling question, was applied as the measure of multiple labeling accuracy (Table 1). As shown by 50 rounds of two-fold random shuffle tests, the hamming loss score is less than 0.3, meaning that at least 70% of binary values in the phenotype matrix are labeled correctly. An alternative approach of accuracy level in the second prediction model is to calculate the exact matches between predicted value and real phenotype. The exact match rate is 7.2~9.3%. The accuracy level is relatively low related to multiple potential factors. The first reason is the limited number of patients with multiple diagnosis, while only 662 patients have more than two diagnosis and 274 patients have more than three diagnoses (Fig. 1c). Therefore, there may not be enough training data for the models to learn from. Secondly, the sample size for some disorders is small, for example, the labeling accuracies for ODD and autism are much lower than other disorders (Tables 2 and 3), meanwhile the sample size for these two are the smallest among all disorders (Fig. 1b). Thirdly, different mental disorders may share genetic risks [28]. Of note, the classification accuracy from random guess for a patient to be correctly classified into one or more of the eight types of disorder is 1/256 (0.4%). In contrast, the labeling from our model is vastly superior and serves as a proof-of-concept that the information could be used to serve as additional references in clinical diagnosis and decision making.

Structural variants in non-coding regions, including UTR, ncRNA, intronic, and intergenic regions, showed no worse prediction abilities than variants in coding regions. However, the weight patterns are different for coding/non-coding variants. The weights of genomic coding variants showed much larger standard deviation than variants in non-coding regions for the two prediction models (Fig. 2). Lack of highly weighted genomic regions (hotspots) for non-coding variants indicates that non-coding variants are likely to function as genomic alternative, instead of causative, compared to coding variants. Also, the weight patterns in 22 chromosomes are highly similar between the two prediction models of coding variants (Fig. 3), but visually different for non-coding variants (Fig. 4). These results indicate that the impact of coding variants are very similar in the eight types of mental disorders, but the regulatory effects from non-coding variants could be essentially different among different disorders.

Enrichment analysis for genes in hotspots, which have weight greater than 1%, was performed (Table 4). The top hotspot at chr19:50000001-55000000 was identified in both categories of stop codon and frameshift SNVs and showed significant enrichment (*p* < 0.05) in genes involving immune response, regulation of transcription/nucleic acid binding, pathways of osteoclast differentiation, and antigen processing/presentation. Previous study reported that schizophrenia, bipolar disorder, and major depression are characterized by several immune-inflammatory alterations outside the brain [29]. In the prediction for mutations on RNA-binding protein target sites, previous results also suggest that binding site dysregulation is a principal contributor to individuals’ risk of developing psychiatric disorders [30]. Osteoporosis was found to co-occur with schizophrenia [31], and auto-antibodies showed higher prevalence in schizophrenia patients’ brain tissues than controls [32]. In addition, another hotspot on chr17:35000001-40000000 contains 33 genes with stop codon SNVs, enriched in chemotaxis biological processes and chemokine activity/signaling pathways. Chemokines were highlighted of novel brain-specific functions and may present novel diagnostic and/or therapeutic targets in psychiatric disorders [33]. Genes in the genomic region at chr11:55000001-60000000 contain stop codon SNVs that are significantly enrichment in G-protein coupled receptor signaling pathway and olfactory transduction. G-protein-coupled receptors were reported to play critical roles in depression, bipolar disorder, and schizophrenia, as well as their treatments [34]. Association has also been reported between olfactory processing and bipolar disorder, major depression, and anxiety [35]. Genes within these hotspots were further explored for potential interactions with FDA-approved medications (Table 5 and Supplementary Table 2). Medications that may be used to treat mental disorders and medications that may cause unwanted drug effects and have supportive animal/clinical evidence are highlighted. For example, *CEPT* interacts with the statin family (e.g., Cerivastatin, Mevastatin, etc.). Previous studies suggested that the adjuvant treatment with a statin may be beneficial for patients with depression and schizophrenia who were prescribed psychotropic drugs [36, 37]. Risperidone, interacting with *TNF*, as an adjunctive therapy for treatment-resistant depression, may improve rate of response and remission based on clinical evidence [38, 39]. *MMP2* interacts with paclitaxel, a commonly used chemotherapy medication, and induces anxiety-like behavior in mouse [40]. Oral dexamethasone for 4 days, which interacts with *SERPINE1*, was significantly more effective than placebo in a randomized, double-blind study of outpatients with depression [41]. Vasopressin, another chemical interacting with *SERPINE1*, was shown to be related to increased risk of stress disorder [42]. Therefore, the hotspots identified in this study may promote the development of treatments/preventions, as well as new drug discoveries, in addition to their roles as biomarkers for the prediction of mental disorders.

**Table 4:** Coding hotspots based on weight of genomic regions and enriched Gene Ontology (GO)/KEGG pathways.

Variation type	Locus	Weight for case–control classifications (%)	Weight for multiple labeling (%)	Num genes contain corresponding variation	Enriched Gene Ontology/pathways (Benjamini–Hochberg adjusted *p* value)
Stop codon	chr19:1-50000000	1.1	1.2	59	–
Stop codon	chr19:50000001-55000000	1.5	1.5	68	Regulation of immune response (2.2E-4); regulation of transcription (5.1E-4); osteoclast differentiation (2.1E-5); antigen processing and presentation (5.2E-4)
Stop codon	chr11:55000001-60000000	1.2	1.2	42	G-protein-coupled receptor signaling pathway (4.3E-27); olfactory receptor activity (2.3E-36); olfactory transduction (1.8E-31)
Stop codon	chr17:35000001-40000000	1.0	1.1	33	Lymphocyte/monocyte chemotaxis (2.1E-3); chemokine-mediated signaling pathway (5.3E-3); chemokine signaling pathway (0.034); cytokine-cytokine receptor interaction (0.036)
Frameshift SNVs	chr11:55000001-60000000	1.3	1.4	49	G-protein-coupled receptor signaling pathway (4E-30); in sensory perception of smell (2E-42); olfactory receptor activity (1.3E-42); olfactory transduction (9.1E-35)
Frameshift SNVs	chr16:55000001-60000000	1.3	1.3	27	–
Frameshift SNVs	chr19:50000001-55000000	1.3	1.4	76	IMMUNE category diseases (6.9E-3); regulation of immune response (3.2E-11); regulation of transcription (3.2E-3); receptor activity (8.3E-3); osteoclast differentiation (1.2E-8); antigen processing and presentation (2.9E-6); natural killer cell-mediated cytotoxicity (5.3E-4)
Frameshift SNVs	chr6:30000001-35000000	1.1	1.2	65	IMMUNE category diseases (2.5E-29); MHC class II receptor activity (1.2E-3); antigen processing and presentation (4.1E-5)
Frameshift SNVs	chr7:100000001-105000000	1.1	1.2	33	–

**Table 5:** Genes in coding hotspots and their interacted medications.

Variation type	Locus	Genes/interacted medications
Frameshift SNVs	chr11:55000001-60000000	TCN1 (cyanocobalamin); MED19 (alcohol)
Frameshift SNVs	chr16:55000001-60000000	SLC12A3 (interacted with 18 medicines); CETP (tamoxifen, atorvastatin, simvastatin, pravastatin, lovastatin, fluvastatin); MMP2 (cyclosporine, pravastatin, bevacizumab, vinblastine, filgrastim, zileuton, paclitaxel, simvastatin, letrozole, streptozocin, acetazolamide, deferoxamine, ramipril)
Frameshift SNVs	chr19:50000001-55000000	KIR2DS4 (methotrexate); KCNC3 (dalfampridine, guanidine hydrochloride); FPR1 (penicillin G potassium, sulfinpyrazone); KLK1 (ecallantide); PRPF31 (metformin); CACNG6 (bepridil hydrochloride, pregabalin, gabapentin enacarbil, gabapentin)
Frameshift SNVs	chr6:30000001-35000000	HCG22 (triamcinolone); CCHCR1 (nevirapine); HSPA1L (carbamazepine); MUCL3 (carboplatin, gemcitabine); EHMT2 (interacted with 189 medications); CDSN (carboplatin, gemcitabine); TCF19 (nevirapine); NOTCH4 (allopurinol); TAPBP (aspirin); ATAT1 (gemcitabine, carboplatin); COL11A2 (ocriplasmin, collagenase clostridium histolyticum); ZBTB22 (aspirin); TNF (interacted with 41 drug)
Frameshift SNVs	chr7:100000001-105000000	SERPINE1 (cetrorelix, hydrochlorothiazide, epirubicin, captopril, orlistat, levothyroxine, nimodipine, dexamethasone, defibrotide, citalopram, urokinase, fluoxetine, vasopressin); STAG3 (vemurafenib); ACHE (interacted with 28 medications); EPHB4 (vandetanib)
Stop codon	chr11:55000001-60000000	TMX2 (alcohol)
Stop codon	chr17:35000001-40000000	SLFN11 (niraparib, temozolomide, talazoparib); CCL3 (infliximab)
Stop codon	chr19:1-5000000	TBXA2R (morphine, iloprost, furosemide, vinblastine, dinoprostone, cyclosporine, aspirin, alprostadil); GRIN3B (felbamate, ketamine hydrochloride, esketamine, amantadine hydrochloride, orphenadrine hydrochloride, acamprosate calcium, orphenadrine, orphenadrine citrate, esketamine hydrochloride, memantine hydrochloride); PLIN3 (galsulfase, idursulfase); AMH (testosterone); MKNK2 (erlotinib, gefitinib, sorafenib); PIP5K1C (alcohol)
Stop codon	chr19:50000001-55000000	KIR2DS4 (methotrexate); KLK4 (ecallantide, bortezomib); CACNG6 (bepridil hydrochloride, pregabalin, gabapentin enacarbil, gabapentin); PRPF31 (metformin); NDUFA3 (metformin hydrochloride)

In summary, our deep learning model showed promising accuracy to differentiate patients versus controls, as well as the potential of labeling patients with multiple disorders. As shown by our study, genetic variants in non-coding regions (e.g., ncRNA, intronic, and intergenic) have comparable labeling capacities to variants in coding regions. However, unlike coding region variants, non-coding variants do not have genomic hotspots and show much more narrow standard deviations, indicating they probably serve as alternative proxy markers. Genes in genomic regions with the highest weights showed enrichment in biological pathways involved in immune responses, antigen/nucleic acid binding, chemokine signaling pathway, and G-protein receptor activities, which with future research may provide mechanistic insights into these mental disorders based on genetic marker support.

## Supplementary information

Supplementary Table 1

Supplementary Table 2

## Data Availability

The data have been uploaded to the database of Genotypes and Phenotypes (dbGaP, https://www.ncbi.nlm.nih.gov/gap/) with the accession number phs001661.v2.p1.
