# Supplementary material for: Application of deep learning algorithm on whole genome sequencing data uncovers structural variants associated with multiple mental disorders in African American patients
Source: Mol Psychiatry. 2022 Jan 8;27(3):1469–78. doi: 10.1038/s41380-021-01418-1 (PMC9095459; doi:10.1038/s41380-021-01418-1)
Supplement: Supplementary file 2 — Supplementary Table 2 [file 41380_2021_1418_MOESM2_ESM.pdf]

Supplementary Table 2. Potential interactions between genes within hotspots with FDA approved medications

Hotspots	gene	drug	interaction types	sources	pmids
frameshift_chr7_100000001-10500	SERPINE1	CETRORELIX		NCI	16391860
frameshift_chr7_100000001-10500	SERPINE1	HYDROCHLOROTHIAZIDE		NCI	11836266
frameshift_chr7_100000001-10500	SERPINE1	EPIRUBICIN		NCI	16444429
frameshift_chr7_100000001-10500	SERPINE1	CAPTOPRIL		NCI	9152782
frameshift_chr7_100000001-10500	SERPINE1	ORLISTAT		NCI	11410822
frameshift_chr7_100000001-10500	SERPINE1	LEVOTHYROXINE		NCI	16075920
frameshift_chr7_100000001-10500	SERPINE1	NIMODIPINE		NCI	11486117
frameshift_chr7_100000001-10500	SERPINE1	DEXAMETHASONE		PharmGKB	18285546
frameshift_chr7_100000001-10500	SERPINE1	DEFIBROTIDE		NCI	12745658
frameshift_chr7_100000001-10500	SERPINE1	CITALOPRAM		PharmGKB	18794724
frameshift_chr7_100000001-10500	SERPINE1	UROKINASE	inducer|substrate	TdgClinicalTrial	12709915|12785198|12745435|12642587|12579271
frameshift_chr7_100000001-10500	SERPINE1	FLUOXETINE		PharmGKB	18794724
frameshift_chr7_100000001-10500	SERPINE1	VASOPRESSIN		NCI	7482412
frameshift_chr7_100000001-10500	STAG3	VEMURAFENIB		CIViC	27500726
frameshift_chr7_100000001-10500	ACHE	PRALIDOXIME	activator|inhibitor	DTC|GuideToPharmacology	19651196|19519385|22206546|21438612|14529460|10801325
frameshift_chr7_100000001-10500	ACHE	AMBENONIUM	inhibitor	TdgClinicalTrial|TEND|TTD	15982786|13679187|2857786|1588924|11752352|11330337|5833399
frameshift_chr7_100000001-10500	ACHE	RIVASTIGMINE	inhibitor	TdgClinicalTrial|ChemblInteractions|TEND|GuideToPharmacology|PharmGKB|TTD	11139819|19470293|18780301|18671661|12636181|19370562|10713582|10673128|11078030|18044073|11752352|10587286|14587496
frameshift_chr7_100000001-10500	ACHE	ECHOTHIOPHATE IODIDE	inhibitor	ChemblInteractions	
frameshift_chr7_100000001-10500	ACHE	PRALIDOXIME CHLORIDE	activator	ChemblInteractions	
frameshift_chr7_100000001-10500	ACHE	BETAMETHASONE		NCI	3896398
frameshift_chr7_100000001-10500	ACHE	NEOSTIGMINE	inhibitor	TdgClinicalTrial|TEND|GuideToPharmacology|TTD	10826417|11313435|12726885|11819669|11752352|12522088|19257799
frameshift_chr7_100000001-10500	ACHE	GALANTAMINE	inhibitor	DTC|TdgClinicalTrial|TEND|GuideToPharmacology|TTD	23540646|10606746|10762042|11172080|12177686|12137632|10971048|11129124|1026294|12481195|11078030|20480924|10592235|11752352|10971049
frameshift_chr7_100000001-10500	ACHE	GALANTAMINE HYDROBROMIDE	inhibitor	ChemblInteractions	
frameshift_chr7_100000001-10500	ACHE	HEXAFLUORENIUM BROMIDE	inhibitor	ChemblInteractions	
frameshift_chr7_100000001-10500	ACHE	TACRINE HYDROCHLORIDE	inhibitor	ChemblInteractions	
frameshift_chr7_100000001-10500	ACHE	RIVASTIGMINE TARTRATE	inhibitor	ChemblInteractions	
frameshift_chr7_100000001-10500	ACHE	PYRIDOSTIGMINE	antagonist|inhibitor	TdgClinicalTrial|TEND|GuideToPharmacology|TTD	10685600|10869589|10669805|11752352|10814558|10366222

frameshift_chr7_100000001-10500	ACHE	ECHOTHIOPHATE	inhibitor	ChemblInteractions|GuideToPharmacology	
frameshift_chr7_100000001-10500	ACHE	EDROPHONIUM CHLORIDE	inhibitor	ChemblInteractions	
frameshift_chr7_100000001-10500	ACHE	DONEPEZIL	inhibitor	DTC|TdgClinicalTrial|TEND|GuideToPharmacology|TTD	20522977|11893059|10368299|20193764|10641971|10466911|10513568|10024872|25282266|11752352
frameshift_chr7_100000001-10500	ACHE	METHADONE		NCI	8149590
frameshift_chr7_100000001-10500	ACHE	TACRINE	inhibitor	DTC|TdgClinicalTrial|TEND|GuideToPharmacology|TTD	15544504|10208549|15544507|10210906|16860785|10513568|10024872|25282266|12871155|10375753|11752352|16880719|17218977
frameshift_chr7_100000001-10500	ACHE	EDROPHONIUM	inhibitor	TdgClinicalTrial|TEND|GuideToPharmacology|TTD	10103097|10089512|19472276|10433700|11752352|10421446|10363282
frameshift_chr7_100000001-10500	ACHE	MALATHION		TTD	
frameshift_chr7_100000001-10500	ACHE	PYRIDOSTIGMINE BROMIDE	inhibitor	ChemblInteractions	
frameshift_chr7_100000001-10500	ACHE	AMBENONIUM CHLORIDE	inhibitor	ChemblInteractions	
frameshift_chr7_100000001-10500	ACHE	THIOGUANINE		NCI	1207869
frameshift_chr7_100000001-10500	ACHE	ISOFLUROPHATE	inhibitor	ChemblInteractions|TTD	12837382|11350858|10378121|10898598|11752352|1692236
frameshift_chr7_100000001-10500	ACHE	DEMECARIUM BROMIDE	inhibitor	ChemblInteractions	
frameshift_chr7_100000001-10500	ACHE	PHYSOSTIGMINE	inhibitor	TdgClinicalTrial|TEND|GuideToPharmacology	10403635|10368402|10873710|11752352|10741458|11141093
frameshift_chr7_100000001-10500	ACHE	NEOSTIGMINE METHYLSULFATE	inhibitor	ChemblInteractions	
frameshift_chr7_100000001-10500	ACHE	DONEPEZIL HYDROCHLORIDE	inhibitor	ChemblInteractions	
frameshift_chr7_100000001-10500	EPHB4	VANDETANIB	inhibitor	ChemblInteractions	
frameshift_chr6_30000001-350000	HCG22	TRIAMCINOLONE		PharmGKB	
frameshift_chr6_30000001-350000	CCHCR1	NEVIRAPINE		PharmGKB	21810746
frameshift_chr6_30000001-350000	HSPA1L	CARBAMAZEPINE		PharmGKB	16538175
frameshift_chr6_30000001-350000	MUCL3	CARBOPLATIN		PharmGKB	31616045
frameshift_chr6_30000001-350000	MUCL3	GEMCITABINE		PharmGKB	31616045
frameshift_chr6_30000001-350000	EHMT2	BISMUTH SUBSALICYLATE		DTC	
frameshift_chr6_30000001-350000	EHMT2	BROMOCRIPTINE MESYLATE		DTC	
frameshift_chr6_30000001-350000	EHMT2	CEFOTAXIME SODIUM		DTC	
frameshift_chr6_30000001-350000	EHMT2	DACTINOMYCIN		DTC	
frameshift_chr6_30000001-350000	EHMT2	ENOXIMONE		DTC	
frameshift_chr6_30000001-350000	EHMT2	BUFEXAMAC		DTC	
frameshift_chr6_30000001-350000	EHMT2	PROMAZINE HYDROCHLORIDE		DTC	
frameshift_chr6_30000001-350000	EHMT2	CYCLOPHOSPHAMIDE		DTC	
frameshift_chr6_30000001-350000	EHMT2	DAUNORUBICIN HYDROCHLORIDE		DTC	
frameshift_chr6_30000001-350000	EHMT2	DEXBROMPHENIRAMINE		DTC	
frameshift_chr6_30000001-350000	EHMT2	DIETHYLCARBAMAZINE CITRATE		DTC	

frameshift_chr6_30000001-350000	EHMT2	DEQUALINIUM CHLORIDE	DTC
frameshift_chr6_30000001-350000	EHMT2	PHYSOSTIGMINE	DTC
frameshift_chr6_30000001-350000	EHMT2	BETAINE HYDROCHLORIDE	DTC
frameshift_chr6_30000001-350000	EHMT2	PALIPERIDONE	DTC
frameshift_chr6_30000001-350000	EHMT2	DYCLONINE	DTC
frameshift_chr6_30000001-350000	EHMT2	TOLAZAMIDE	DTC
frameshift_chr6_30000001-350000	EHMT2	FLUTAMIDE	DTC
frameshift_chr6_30000001-350000	EHMT2	CEFACLOR	DTC
frameshift_chr6_30000001-350000	EHMT2	CHLOROXINE	DTC
frameshift_chr6_30000001-350000	EHMT2	MELPHALAN	DTC
frameshift_chr6_30000001-350000	EHMT2	RABEPRAZOLE	DTC
frameshift_chr6_30000001-350000	EHMT2	CLOFAZIMINE	DTC
frameshift_chr6_30000001-350000	EHMT2	RESCINNAMINE	DTC
frameshift_chr6_30000001-350000	EHMT2	NIMESULIDE	DTC
frameshift_chr6_30000001-350000	EHMT2	CAPTOPRIL	DTC
frameshift_chr6_30000001-350000	EHMT2	ALITRETINOIN	DTC
frameshift_chr6_30000001-350000	EHMT2	DIPYRIDAMOLE	DTC
frameshift_chr6_30000001-350000	EHMT2	FLUPHENAZINE	DTC
frameshift_chr6_30000001-350000	EHMT2	PYRITHIONE	DTC
frameshift_chr6_30000001-350000	EHMT2	LEVONORDEFRIN	DTC
frameshift_chr6_30000001-350000	EHMT2	PAROXETINE	DTC
frameshift_chr6_30000001-350000	EHMT2	SULFAPYRIDINE	DTC
frameshift_chr6_30000001-350000	EHMT2	TRIPROLIDINE	DTC
frameshift_chr6_30000001-350000	EHMT2	MIFEPRISTONE	DTC
frameshift_chr6_30000001-350000	EHMT2	HISTAMINE	DTC
frameshift_chr6_30000001-350000	EHMT2	SERTACONAZOLE NITRATE	DTC
frameshift_chr6_30000001-350000	EHMT2	OXITRIPTAN	DTC
frameshift_chr6_30000001-350000	EHMT2	PROPRANOLOL	DTC
frameshift_chr6_30000001-350000	EHMT2	DOXORUBICIN HYDROCHLORIDE	DTC
frameshift_chr6_30000001-350000	EHMT2	ORPHENADRINE CITRATE	DTC
frameshift_chr6_30000001-350000	EHMT2	PAPAVERINE	DTC
frameshift_chr6_30000001-350000	EHMT2	PHENELZINE SULFATE	DTC
frameshift_chr6_30000001-350000	EHMT2	ISOETHARINE	DTC
frameshift_chr6_30000001-350000	EHMT2	HYDROXYZINE PAMOATE	DTC
frameshift_chr6_30000001-350000	EHMT2	IODOFORM	DTC
frameshift_chr6_30000001-350000	EHMT2	RANITIDINE	DTC
frameshift_chr6_30000001-350000	EHMT2	AMANTADINE HYDROCHLORIDE	DTC
frameshift_chr6_30000001-350000	EHMT2	ALFUZOSIN	DTC
frameshift_chr6_30000001-350000	EHMT2	SALMETEROL XINAFOATE	DTC
frameshift_chr6_30000001-350000	EHMT2	TRETINOIN	DTC
frameshift_chr6_30000001-350000	EHMT2	CYCLOSERINE	DTC
frameshift_chr6_30000001-350000	EHMT2	CHLOROQUINE	DTC
frameshift_chr6_30000001-350000	EHMT2	KETOCONAZOLE	DTC
frameshift_chr6_30000001-350000	EHMT2	RAZOXANE	DTC
frameshift_chr6_30000001-350000	EHMT2	ETHAMSYLATE	DTC
frameshift_chr6_30000001-350000	EHMT2	NICLOSAMIDE	DTC

frameshift_chr6_30000001-350000	EHMT2	APAZONE	DTC
frameshift_chr6_30000001-350000	EHMT2	RALOXIFENE HYDROCHLORIDE	DTC
frameshift_chr6_30000001-350000	EHMT2	ERGOCALCIFEROL	DTC
frameshift_chr6_30000001-350000	EHMT2	PIRENZEPINE	DTC
frameshift_chr6_30000001-350000	EHMT2	CYTARABINE	DTC
frameshift_chr6_30000001-350000	EHMT2	CEFOTAXIME	DTC
frameshift_chr6_30000001-350000	EHMT2	NISOLDIPINE	DTC
frameshift_chr6_30000001-350000	EHMT2	BRIMONIDINE	DTC
frameshift_chr6_30000001-350000	EHMT2	DOXYLAMINE	DTC
frameshift_chr6_30000001-350000	EHMT2	LANSOPRAZOLE	DTC
frameshift_chr6_30000001-350000	EHMT2	MOLINDONE	DTC
frameshift_chr6_30000001-350000	EHMT2	FENOLDOPAM	DTC
frameshift_chr6_30000001-350000	EHMT2	FLUNARIZINE	DTC
frameshift_chr6_30000001-350000	EHMT2	BEPRIDIL	DTC
frameshift_chr6_30000001-350000	EHMT2	THEOPHYLLINE	DTC
frameshift_chr6_30000001-350000	EHMT2	TELMISARTAN	DTC
frameshift_chr6_30000001-350000	EHMT2	ISOETHARINE MESYLATE	DTC
frameshift_chr6_30000001-350000	EHMT2	CEFOTIAM HYDROCHLORIDE	DTC
frameshift_chr6_30000001-350000	EHMT2	DOXYCYCLINE	DTC
frameshift_chr6_30000001-350000	EHMT2	DEFERIPRONE	DTC
frameshift_chr6_30000001-350000	EHMT2	DOPAMINE HYDROCHLORIDE	DTC
frameshift_chr6_30000001-350000	EHMT2	CYCLOPHOSPHAMIDE	DTC
frameshift_chr6_30000001-350000	EHMT2	NIFEDIPINE	DTC
frameshift_chr6_30000001-350000	EHMT2	MAPROTILINE HYDROCHLORIDE	DTC
frameshift_chr6_30000001-350000	EHMT2	METHIMAZOLE	DTC
frameshift_chr6_30000001-350000	EHMT2	MESALAMINE	DTC
frameshift_chr6_30000001-350000	EHMT2	CEPHALOTHIN	DTC
frameshift_chr6_30000001-350000	EHMT2	HYDRALAZINE	DTC
frameshift_chr6_30000001-350000	EHMT2	GALLAMINE	DTC
frameshift_chr6_30000001-350000	EHMT2	DOPAMINE	DTC
frameshift_chr6_30000001-350000	EHMT2	AMILORIDE	DTC
frameshift_chr6_30000001-350000	EHMT2	DIMERCAPROL	DTC
frameshift_chr6_30000001-350000	EHMT2	CEPHALEXIN	DTC
frameshift_chr6_30000001-350000	EHMT2	OMEPRAZOLE MAGNESIUM	DTC
frameshift_chr6_30000001-350000	EHMT2	TRIFLUOPERAZINE	DTC
frameshift_chr6_30000001-350000	EHMT2	OXYMETHOLONE	DTC
frameshift_chr6_30000001-350000	EHMT2	BUSPIRONE	DTC
frameshift_chr6_30000001-350000	EHMT2	CEFAZOLIN	DTC
frameshift_chr6_30000001-350000	EHMT2	ESTRADIOL	DTC
frameshift_chr6_30000001-350000	EHMT2	CYSTEAMINE HYDROCHLORIDE	DTC
frameshift_chr6_30000001-350000	EHMT2	ETHACRYNIC ACID	DTC
frameshift_chr6_30000001-350000	EHMT2	METHOTREXATE	DTC
frameshift_chr6_30000001-350000	EHMT2	NALOXONE HYDROCHLORIDE	DTC
frameshift_chr6_30000001-350000	EHMT2	THIOTEPA	DTC

frameshift_chr6_30000001-350000	EHMT2	METHYSERGIDE	DTC
frameshift_chr6_30000001-350000	EHMT2	PROCHLORPERAZINE EDISYLATE	DTC
frameshift_chr6_30000001-350000	EHMT2	LEVODOPA	DTC
frameshift_chr6_30000001-350000	EHMT2	EFLORNITHINE	DTC
frameshift_chr6_30000001-350000	EHMT2	HYDROQUINONE	DTC
frameshift_chr6_30000001-350000	EHMT2	METERGOLINE	DTC
frameshift_chr6_30000001-350000	EHMT2	ETHOPROPAZINE	DTC
frameshift_chr6_30000001-350000	EHMT2	ANTHRALIN	DTC
frameshift_chr6_30000001-350000	EHMT2	PROFLAVINE	DTC
frameshift_chr6_30000001-350000	EHMT2	ADEFOVIR DIPIVOXIL	DTC
frameshift_chr6_30000001-350000	EHMT2	PAROXETINE HYDROCHLORIDE	DTC
frameshift_chr6_30000001-350000	EHMT2	NALIDIXIC ACID	DTC
frameshift_chr6_30000001-350000	EHMT2	MIANSERIN HYDROCHLORIDE	DTC
frameshift_chr6_30000001-350000	EHMT2	CYCLOBENZAPRINE HYDROCHLORIDE	DTC
frameshift_chr6_30000001-350000	EHMT2	CITALOPRAM	DTC
frameshift_chr6_30000001-350000	EHMT2	PHENELZINE	DTC
frameshift_chr6_30000001-350000	EHMT2	PROMETHAZINE	DTC
frameshift_chr6_30000001-350000	EHMT2	BETAXOLOL HYDROCHLORIDE	DTC
frameshift_chr6_30000001-350000	EHMT2	GUANFACINE	DTC
frameshift_chr6_30000001-350000	EHMT2	SULFINPYRAZONE	DTC
frameshift_chr6_30000001-350000	EHMT2	GLUTAMIC ACID HYDROCHLORIDE	DTC
frameshift_chr6_30000001-350000	EHMT2	TAMOXIFEN	DTC
frameshift_chr6_30000001-350000	EHMT2	MENADIONE	DTC
frameshift_chr6_30000001-350000	EHMT2	METHYLERGONOVINE	DTC
frameshift_chr6_30000001-350000	EHMT2	DIAZOXIDE	DTC
frameshift_chr6_30000001-350000	EHMT2	DISULFIRAM	DTC
frameshift_chr6_30000001-350000	EHMT2	IMIPRAMINE	DTC
frameshift_chr6_30000001-350000	EHMT2	CARBIDOPA	DTC
frameshift_chr6_30000001-350000	EHMT2	TAMOXIFEN CITRATE	DTC
frameshift_chr6_30000001-350000	EHMT2	BROMOCRIPTINE	DTC
frameshift_chr6_30000001-350000	EHMT2	APOMORPHINE	DTC
frameshift_chr6_30000001-350000	EHMT2	BENSERAZIDE	DTC
frameshift_chr6_30000001-350000	EHMT2	OMEPRAZOLE	DTC
frameshift_chr6_30000001-350000	EHMT2	LOVASTATIN	DTC
frameshift_chr6_30000001-350000	EHMT2	OXYTETRACYCLINE	DTC
frameshift_chr6_30000001-350000	EHMT2	EPINEPHRINE BITARTRATE	DTC
frameshift_chr6_30000001-350000	EHMT2	AMSACRINE	DTC
frameshift_chr6_30000001-350000	EHMT2	MITOXANTRONE HYDROCHLORIDE	DTC
frameshift_chr6_30000001-350000	EHMT2	METHYLDOPA	DTC
frameshift_chr6_30000001-350000	EHMT2	INAMRINONE	DTC
frameshift_chr6_30000001-350000	EHMT2	METHAZOLAMIDE	DTC
frameshift_chr6_30000001-350000	EHMT2	CYSTEAMINE	DTC
frameshift_chr6_30000001-350000	EHMT2	DYCLONINE HYDROCHLORIDE	DTC

frameshift_chr6_30000001-350000	EHMT2	CEPHAPIRIN SODIUM	DTC
frameshift_chr6_30000001-350000	EHMT2	CEFOTETAN	DTC
frameshift_chr6_30000001-350000	EHMT2	DEMECLOCYCLINE HYDROCHLORIDE	DTC
frameshift_chr6_30000001-350000	EHMT2	CHLORPROMAZINE HYDROCHLORIDE	DTC
frameshift_chr6_30000001-350000	EHMT2	PROCHLORPERAZINE	DTC
frameshift_chr6_30000001-350000	EHMT2	DIACEREIN	DTC
frameshift_chr6_30000001-350000	EHMT2	PIRETANIDE	DTC
frameshift_chr6_30000001-350000	EHMT2	NICARDIPINE	DTC
frameshift_chr6_30000001-350000	EHMT2	NORFLOXACIN	DTC
frameshift_chr6_30000001-350000	EHMT2	GALLAMINE TRIETHIODIDE	DTC
frameshift_chr6_30000001-350000	EHMT2	CISPLATIN	DTC
frameshift_chr6_30000001-350000	EHMT2	CEPHALOTHIN SODIUM	DTC
frameshift_chr6_30000001-350000	EHMT2	EPHEDRINE SULFATE	DTC
frameshift_chr6_30000001-350000	EHMT2	FOMEPIZOLE	DTC
frameshift_chr6_30000001-350000	EHMT2	MITOXANTRONE	DTC
frameshift_chr6_30000001-350000	EHMT2	THIOGUANINE	DTC
frameshift_chr6_30000001-350000	EHMT2	FLUSPIRILENE	DTC
frameshift_chr6_30000001-350000	EHMT2	SULFADIAZINE, SILVER	DTC
frameshift_chr6_30000001-350000	EHMT2	NOREPINEPHRINE	DTC
frameshift_chr6_30000001-350000	EHMT2	CHOLECALCIFEROL	DTC
frameshift_chr6_30000001-350000	EHMT2	CLOMETHIAZOLE	DTC
frameshift_chr6_30000001-350000	EHMT2	KETANSERIN	DTC
frameshift_chr6_30000001-350000	EHMT2	RISEDRONIC ACID	DTC
frameshift_chr6_30000001-350000	EHMT2	DICYCLOMINE	DTC
frameshift_chr6_30000001-350000	EHMT2	ISOPROTERENOL	DTC
frameshift_chr6_30000001-350000	EHMT2	ZIPRASIDONE	DTC
frameshift_chr6_30000001-350000	EHMT2	PYROGALLOL	DTC
frameshift_chr6_30000001-350000	EHMT2	VINPOCETINE	DTC
frameshift_chr6_30000001-350000	EHMT2	CYSTEINE	DTC
frameshift_chr6_30000001-350000	EHMT2	DILTIAZEM HYDROCHLORIDE	DTC
frameshift_chr6_30000001-350000	EHMT2	PHENINDIONE	DTC
frameshift_chr6_30000001-350000	EHMT2	NICARDIPINE HYDROCHLORIDE	DTC
frameshift_chr6_30000001-350000	EHMT2	SULINDAC	DTC
frameshift_chr6_30000001-350000	EHMT2	PROMETHAZINE HYDROCHLORIDE	DTC
frameshift_chr6_30000001-350000	EHMT2	DEBRISOQUIN SULFATE	DTC
frameshift_chr6_30000001-350000	EHMT2	LOMEFLOXACIN	DTC
frameshift_chr6_30000001-350000	EHMT2	TANNIC ACID	DTC
frameshift_chr6_30000001-350000	EHMT2	TOLFENAMIC ACID	DTC
frameshift_chr6_30000001-350000	EHMT2	STREPTOMYCIN	DTC
frameshift_chr6_30000001-350000	EHMT2	ISOCONAZOLE	DTC
frameshift_chr6_30000001-350000	EHMT2	IDARUBICIN	DTC
frameshift_chr6_30000001-350000	EHMT2	MESORIDAZINE	DTC
frameshift_chr6_30000001-350000	EHMT2	DAUNORUBICIN	DTC
frameshift_chr6_30000001-350000	EHMT2	TRIMETREXATE	DTC

frameshift_chr6_30000001-350000	EHMT2	YOHIMBINE		DTC	
frameshift_chr6_30000001-350000	CDSN	CARBOPLATIN		PharmGKB	31616045
frameshift_chr6_30000001-350000	CDSN	GEMCITABINE		PharmGKB	31616045
frameshift_chr6_30000001-350000	TCF19	NEVIRAPINE		PharmGKB	21810746
frameshift_chr6_30000001-350000	NOTCH4	ALLOPURINOL		PharmGKB	29193002
frameshift_chr6_30000001-350000	TAPBP	ASPIRIN		PharmGKB	23736108
frameshift_chr6_30000001-350000	ATAT1	GEMCITABINE		PharmGKB	31616045
frameshift_chr6_30000001-350000	ATAT1	CARBOPLATIN		PharmGKB	31616045
frameshift_chr6_30000001-350000	COL11A2	OCRIPLASMIN		ChemblInteractions	
frameshift_chr6_30000001-350000	COL11A2	COLLAGENASE CLOSTRIDIUM HISTOLYTICUM		ChemblInteractions	
frameshift_chr6_30000001-350000	ZBTB22	ASPIRIN		PharmGKB	
frameshift_chr6_30000001-350000	TNF	CARBAMAZEPINE		NCI|PharmGKB	15565432|11294926
frameshift_chr6_30000001-350000	TNF	CEFOTAXIME		NCI	8354907|10989981
frameshift_chr6_30000001-350000	TNF	PYRIDOXINE		NCI	16277693
frameshift_chr6_30000001-350000	TNF	AMPHOTERICIN B		DTC	
frameshift_chr6_30000001-350000	TNF	MILTEFOSINE		NCI	7883777
frameshift_chr6_30000001-350000	TNF	RIFAMPIN		PharmGKB	22151084
frameshift_chr6_30000001-350000	TNF	PROPYLTHIOURACIL		NCI	15119959
frameshift_chr6_30000001-350000	TNF	METHIMAZOLE		NCI	8491516
frameshift_chr6_30000001-350000	TNF	HYDROXYCHLOROQUINE		NCI	9002011
frameshift_chr6_30000001-350000	TNF	ETHAMBUTOL		PharmGKB	22151084
frameshift_chr6_30000001-350000	TNF	MEROPENEM		NCI	8354907
frameshift_chr6_30000001-350000	TNF	DIGOXIN		DTC	
frameshift_chr6_30000001-350000	TNF	GEMCITABINE		PharmGKB	31616045
frameshift_chr6_30000001-350000	TNF	ADALIMUMAB	antibody|inhibitor	TdgClinicalTrial|ChemblInteractions|TEND|PharmGKB|TTD	12044041|16720636|16909270|23057546|15022409|26244882|12847678|18050183|18713756|19365401|22760475|24192118|14532145|17343250|22960943|15046527|22129793|11752352|12190096|18438841|15200343
frameshift_chr6_30000001-350000	TNF	INSULIN		NCI	16125526|9287059
frameshift_chr6_30000001-350000	TNF	CYCLOSPORINE		PharmGKB	18444945
frameshift_chr6_30000001-350000	TNF	ALTEPLASE		NCI	8615653
frameshift_chr6_30000001-350000	TNF	PENTOXIFYLLINE		TTD	
frameshift_chr6_30000001-350000	TNF	THALIDOMIDE	inhibitor	TdgClinicalTrial|TEND|TTD	8755512|12046682|12167383|12105857|12102294|11752352|12113124
frameshift_chr6_30000001-350000	TNF	LACTULOSE		NCI	11226652
frameshift_chr6_30000001-350000	TNF	RABEPRAZOLE		NCI	16815316
frameshift_chr6_30000001-350000	TNF	LENALIDOMIDE		ClearityFoundationClinicalTrial|TTD	
frameshift_chr6_30000001-350000	TNF	STAVUDINE		PharmGKB	20887379
frameshift_chr6_30000001-350000	TNF	DIDANOSINE		NCI	9430255
frameshift_chr6_30000001-350000	TNF	SORAFENIB		PharmGKB	22736425
frameshift_chr6_30000001-350000	TNF	GLIMEPIRIDE		NCI	14686960

frameshift_chr6_30000001-350000	TNF	MIDAZOLAM		NCI	16406030
frameshift_chr6_30000001-350000	TNF	BUPIVACAINE		NCI	15781526
frameshift_chr6_30000001-350000	TNF	GOLIMUMAB	inhibitor|antibody	TdgClinicalTrial|ChemblInteractions|TEND|TTD	21079302
frameshift_chr6_30000001-350000	TNF	GENTAMICIN		NCI	14565862
frameshift_chr6_30000001-350000	TNF	SPIRONOLACTONE		NCI	16837769
frameshift_chr6_30000001-350000	TNF	ETANERCEPT	inhibitor|antibody	TdgClinicalTrial|ChemblInteractions|TEND|PharmGKB|TTD	16720636|16909270|23057546|26244882|12847678|18050183|18713756|19365401|22760475|24192118|17343250|22960943|10375846|10405518|10206649|22129793|11752352|12190096|10338381|18438841|10357816
frameshift_chr6_30000001-350000	TNF	ISONIAZID		PharmGKB	22151084
frameshift_chr6_30000001-350000	TNF	CERTOLIZUMAB PEGOL	inhibitor	ChemblInteractions	
frameshift_chr6_30000001-350000	TNF	METHYLENE BLUE		DTC	
frameshift_chr6_30000001-350000	TNF	ATORVASTATIN		PharmGKB	18997459
frameshift_chr6_30000001-350000	TNF	INFLIXIMAB	inhibitor	TdgClinicalTrial|ChemblInteractions|TEND|PharmGKB|TTD	16720636|16909270|23057546|16456024|26244882|12847678|18050183|18713756|19365401|22760475|16052578|24192118|12110154|17343250|22960943|15481318|15691299|15674127|15804598|22129793|11752352|25311255|12190096|18438841|15691217|17673491|15695296|17642244|16622728
frameshift_chr6_30000001-350000	TNF	CARBOPLATIN		PharmGKB	31616045
frameshift_chr6_30000001-350000	TNF	OMEPRAZOLE		NCI	16815316
frameshift_chr6_30000001-350000	TNF	RISPERIDONE		NCI	15567770|11545247
frameshift_chr6_30000001-350000	TNF	PYRAZINAMIDE		PharmGKB	22151084
frameshift_chr11_55000001-60000	TCN1	CYANOCOBALAMIN		NCI	2876185
frameshift_chr11_55000001-60000	MED19	ALCOHOL		PharmGKB	32451486
frameshift_chr16_55000001-60000	SLC12A3	INDAPAMIDE	inhibitor	ChemblInteractions	24444254|23729436|20528637
frameshift_chr16_55000001-60000	SLC12A3	POLYTHIAZIDE	inhibitor	TdgClinicalTrial|ChemblInteractions|TEND|TTD	10894798|19474192|12217863|11752352
frameshift_chr16_55000001-60000	SLC12A3	TORSEMIDE		PharmGKB	20877298|17460608
frameshift_chr16_55000001-60000	SLC12A3	METHYCLOTHIAZIDE	inhibitor	ChemblInteractions	
frameshift_chr16_55000001-60000	SLC12A3	HYDROFLUMETHIAZIDE	inhibitor	ChemblInteractions	
frameshift_chr16_55000001-60000	SLC12A3	FUROSEMIDE		PharmGKB	20877298|17460608
frameshift_chr16_55000001-60000	SLC12A3	BUMETANIDE		PharmGKB	20877298|17460608
frameshift_chr16_55000001-60000	SLC12A3	DIAZOXIDE		TdgClinicalTrial|TEND	17139284|17016423
frameshift_chr16_55000001-60000	SLC12A3	METOLAZONE	inhibitor	TdgClinicalTrial|ChemblInteractions|TEND|GuideToPharmacology|TTD	11752352

frameshift_chr16_55000001-60000	SLC12A3	CHLOROTHIAZIDE SODIUM	inhibitor	ChemblInteractions	
frameshift_chr16_55000001-60000	SLC12A3	CHLORTHALIDONE	inhibitor	ChemblInteractions	
frameshift_chr16_55000001-60000	SLC12A3	TRICHLORMETHIAZIDE	inhibitor	ChemblInteractions	17414671|20187262|11752352|14610216
frameshift_chr16_55000001-60000	SLC12A3	QUINETHAZONE	inhibitor	TdgClinicalTrial|ChemblInteractions|TEND	19474192|19636250|12538756|20528637
frameshift_chr16_55000001-60000	SLC12A3	HYDROCHLOROTHIAZIDE	inhibitor	TdgClinicalTrial|ChemblInteractions|TEND|GuideToPharmacology	10894798|12772080|16078592|16172412|9038817|11752352|9596079
frameshift_chr16_55000001-60000	SLC12A3	BENZTHIAZIDE	inhibitor	TdgClinicalTrial|ChemblInteractions|TEND	17139284|12538756|17016423|31285285
frameshift_chr16_55000001-60000	SLC12A3	CHLOROTHIAZIDE	inhibitor	TdgClinicalTrial|ChemblInteractions|TEND|GuideToPharmacology	15069170|12515852|15283765|11014932|11456284
frameshift_chr16_55000001-60000	SLC12A3	CYCLOTHIAZIDE	inhibitor	ChemblInteractions|GuideToPharmacology	
frameshift_chr16_55000001-60000	SLC12A3	BENDROFLUMETHIAZIDE	inhibitor	TdgClinicalTrial|ChemblInteractions|TEND|TTD	10894798|18216144|11752352
frameshift_chr16_55000001-60000	CETP	TAMOXIFEN		NCI	9751231
frameshift_chr16_55000001-60000	CETP	ATORVASTATIN		PharmGKB	16103896|12663600|15856070
frameshift_chr16_55000001-60000	CETP	SIMVASTATIN		PharmGKB	17931083|15856070
frameshift_chr16_55000001-60000	CETP	PRAVASTATIN		PharmGKB	18957472|14660992|15044381|9420339|15856070
frameshift_chr16_55000001-60000	CETP	LOVASTATIN		PharmGKB	16103896
frameshift_chr16_55000001-60000	CETP	FLUVASTATIN		PharmGKB	16002074
frameshift_chr16_55000001-60000	MMP2	CYCLOSPORINE		NCI	12639820
frameshift_chr16_55000001-60000	MMP2	PRAVASTATIN		NCI	15842807
frameshift_chr16_55000001-60000	MMP2	BEVACIZUMAB		CIViC	26921265
frameshift_chr16_55000001-60000	MMP2	VINBLASTINE		NCI	10590059
frameshift_chr16_55000001-60000	MMP2	FILGRASTIM		NCI	11391619
frameshift_chr16_55000001-60000	MMP2	ZILEUTON		DTC	24074025
frameshift_chr16_55000001-60000	MMP2	PACLITAXEL		NCI	9174131
frameshift_chr16_55000001-60000	MMP2	SIMVASTATIN		NCI	16436088
frameshift_chr16_55000001-60000	MMP2	LETROZOLE		NCI	12569569
frameshift_chr16_55000001-60000	MMP2	STREPTOZOCIN		NCI	9394952
frameshift_chr16_55000001-60000	MMP2	ACETAZOLAMIDE		DTC	24074025
frameshift_chr16_55000001-60000	MMP2	DEFEROXAMINE		DTC	24074025
frameshift_chr16_55000001-60000	MMP2	RAMIPRIL		NCI	16166267
frameshift_chr19_50000001-55000	KIR2DS4	METHOTREXATE		PharmGKB	25069714
frameshift_chr19_50000001-55000	KCNC3	DALFAMPRIDINE	antagonist|blocker	ChemblInteractions	16472864
frameshift_chr19_50000001-55000	KCNC3	GUANIDINE HYDROCHLORIDE	blocker	ChemblInteractions	
frameshift_chr19_50000001-55000	FPR1	PENICILLIN G POTASSIUM		DTC	
frameshift_chr19_50000001-55000	FPR1	SULFINPYRAZONE		DTC	
frameshift_chr19_50000001-55000	KLK1	ECALLANTIDE		TdgClinicalTrial|TEND	
frameshift_chr19_50000001-55000	PRPF31	METFORMIN		PharmGKB	29650774

frameshift_chr19_50000001-55000	CACNG6	BEPRIDIL HYDROCHLORIDE	blocker	ChemblInteractions	
frameshift_chr19_50000001-55000	CACNG6	PREGABALIN	modulator	ChemblInteractions	
frameshift_chr19_50000001-55000	CACNG6	GABAPENTIN ENACARBIL	modulator	ChemblInteractions	
frameshift_chr19_50000001-55000	CACNG6	GABAPENTIN	modulator	ChemblInteractions	
stop_chr11_55000001-60000000	TMX2	ALCOHOL		PharmGKB	32451486
stop_chr17_35000001-40000000	ERBB2	DASATINIB		DTC	
stop_chr17_35000001-40000000	ERBB2	TALAZOPARIB		PharmGKB|FDA	
stop_chr17_35000001-40000000	ERBB2	ANASTROZOLE		CGI	
stop_chr17_35000001-40000000	ERBB2	LAPATINIB DITOSYLATE	inhibitor	ChemblInteractions	
stop_chr17_35000001-40000000	ERBB2	PALBOCICLIB		JAX-CKB|CGI|PharmGKB|FDA	27020857|19874578|25221644
stop_chr17_35000001-40000000	ERBB2	DOCETAXEL		JAX-CKB|CIViC|PharmGKB	30452336|22149875|23801166|27052654|25693012|30071039|25185099
stop_chr17_35000001-40000000	ERBB2	TRIFLURIDINE		FDA	
stop_chr17_35000001-40000000	ERBB2	SUNITINIB		JAX-CKB	24606768
stop_chr17_35000001-40000000	ERBB2	FILGRASTIM		NCI	16549824
stop_chr17_35000001-40000000	ERBB2	CYCLOPHOSPHAMIDE		JAX-CKB	
stop_chr17_35000001-40000000	ERBB2	EPIRUBICIN		NCI	11759828
stop_chr17_35000001-40000000	ERBB2	GEFITINIB	inhibitor	DTC|JAX-CKB|NCI|MyCancerGenomeClinicalTrial|PharmGKB	26545934|23220880|17638894|20151670|16818711
stop_chr17_35000001-40000000	ERBB2	OSIMERTINIB MESYLATE	inhibitor	ChemblInteractions	
stop_chr17_35000001-40000000	ERBB2	EXEMESTANE		JAX-CKB|CGI	
stop_chr17_35000001-40000000	ERBB2	ERIBULIN		PharmGKB|FDA	
stop_chr17_35000001-40000000	ERBB2	GEMCITABINE		JAX-CKB|NCI	15581051
stop_chr17_35000001-40000000	ERBB2	CAPECITABINE		JAX-CKB|CIViC	20504363|17192538|17679724|19289619|25287822|26432108|26487584|26920887
stop_chr17_35000001-40000000	ERBB2	VANDETANIB	inhibitor	ChemblInteractions	
stop_chr17_35000001-40000000	ERBB2	CISPLATIN		JAX-CKB	
stop_chr17_35000001-40000000	ERBB2	NERATINIB	inhibitor	TALC|MyCancerGenome|TdgClinicalTrial|JAX-CKB|ChemblInteractions|CGI|DoCM|CIViC|GuideToPharmacology|MyCancerGenomeClinicalTrial|PharmGKB|TTD|FDA|OncoKB	26619011|28274957|24516025|28679771|23632474|22908275|24009064|29523624|26874901|23953056|28363995|26333383|27078022|28539475|22418700|29146401|20142587|25287822|24323026|25157968|22046346|23220880|16397024|27697991|26243863
stop_chr17_35000001-40000000	ERBB2	FLUOROURACIL		JAX-CKB	28784859|27626067|26432108

stop_chr17_35000001-40000000	ERBB2	TRASTUZUMAB	antibody|inhibitor	TALC|ClearityFoundationBiomarkers|MyCancerGenome|TdgClinicalTrial|ClearityFoundationClinicalTrial|JAX-CKB|ChemblInteractions|NCI|CGI|DoCM|CIViC|GuideToPharmacology|PharmGKB|TTD|FDA|OncoKB	27900589|26619011|20504363|16091755|31453370|17936563|22257673|19840887|2013|21676217|26759238|30452336|26598547|24470511|28784859|27140927|11248153|27108243|22908275|21107682|18555254|26880266|15236790|26296355|22149875|23801166|29523624|26469692|26874901|22850551|24691421|25601188|24606768|24982373|26104654|17660958|20728210|11098307|20975068|17679724|27626067|23948973|26099744|28167203|30857956|23940356|19289619|23204226|24742739|28539475|15911866|26245675|29320312|25693012|29146401|11752352|19874578|20003286|9788323|25157968|30071039|2605|23220880|21558396|18690878|25370464|26432108|16397024|24300914|29584549|22493419|27697991|26243863|16596213|12525520|17693647|16236737|20124187
stop_chr17_35000001-40000000	ERBB2	ALPELISIB		PharmGKB|FDA	
stop_chr17_35000001-40000000	ERBB2	CETUXIMAB		JAX-CKB|CIViC	22586653|26296355|21900593|23948973|28223103|26243863
stop_chr17_35000001-40000000	ERBB2	CARBOPLATIN		CIViC|PharmGKB	30071039|29584549
stop_chr17_35000001-40000000	ERBB2	PANITUMUMAB		JAX-CKB	26243863
stop_chr17_35000001-40000000	ERBB2	PERTUZUMAB	inhibitor|antibody	TALC|ClearityFoundationBiomarkers|MyCancerGenome|TdgClinicalTrial|JAX-CKB|ChemblInteractions|CGI|CIViC|GuideToPharmacology|PharmGKB|TTD|FDA|OncoKB	31453370|22586653|22149875|23801166|30857956|23940356|27052654|28539475|29320312|25693012|15093539
stop_chr17_35000001-40000000	ERBB2	IBRUTINIB	inhibitor	JAX-CKB|CIViC|GuideToPharmacology	27256378|28830912
stop_chr17_35000001-40000000	ERBB2	RIBOCICLIB		PharmGKB|FDA	
stop_chr17_35000001-40000000	ERBB2	CHLORAMBUCIL		NCI	11691913
stop_chr17_35000001-40000000	ERBB2	LEUCOVORIN		JAX-CKB	28784859|27626067|26432108
stop_chr17_35000001-40000000	ERBB2	TRASTUZUMAB EMTANSINE	inhibitor	ChemblInteractions	
stop_chr17_35000001-40000000	ERBB2	OSIMERTINIB		JAX-CKB|CGI	27252416
stop_chr17_35000001-40000000	ERBB2	GANCICLOVIR		NCI	8986436

stop_chr17_35000001-40000000	ERBB2	TEMSIROLIMUS		JAX-CKB	24323026
stop_chr17_35000001-40000000	ERBB2	HYDROCORTISONE		NCI	7909787
stop_chr17_35000001-40000000	ERBB2	METFORMIN		JAX-CKB	28375706
stop_chr17_35000001-40000000	ERBB2	LETROZOLE		JAX-CKB|CGI	19786658
stop_chr17_35000001-40000000	ERBB2	TAMOXIFEN		NCI|CGI	11916237|15901136|17266042
stop_chr17_35000001-40000000	ERBB2	MASOPROCOL		TTD	
stop_chr17_35000001-40000000	ERBB2	RAMUCIRUMAB		JAX-CKB	25185099
stop_chr17_35000001-40000000	ERBB2	BEVACIZUMAB		JAX-CKB	23940356|26432108
stop_chr17_35000001-40000000	ERBB2	AFATINIB	inhibitor	TALC|MyCancerGenome|TdgClinicalTrial|JAX-CKB|CGI|CIViC|GuideToPharmacology|MyCancerGenomeClinicalTrial	19122144|28274957|26545934|27044931|23632474|24971884|22325357|27923043|26296355|25589492|23775486|28363995|23578997|26964772|26559459|23948973|30096481|28167203|22418700|20142587|25370464|26243863|21617858|26596672
stop_chr17_35000001-40000000	ERBB2	EVEROLIMUS		JAX-CKB|CGI|PharmGKB|FDA	21107682|20975068|24742739
stop_chr17_35000001-40000000	ERBB2	LAPATINIB	antagonist|inhibitor	TALC|DTC|ClearityFoundationBiomarkers|MyCancerGenome|TdgClinicalTrial|JAX-CKB|CGI|TEND|DoCM|CIViC|GuideToPharmacology|PharmGKB|TTD|FDA|OncoKB	26619011|25305330|17192538|20179222|16091755|27595477|12214266|24516025|22257673|20110044|18413839|27197158|27140927|15374980|26270481|27108243|22908275|24971884|14633707|22586653|27760111|26296355|26570998|2015169|18774637|27450453|24355130|16894399|27900369|23948973|14751502|27026198|23940356|25398453|24868024|26245675|19786658|26811533|11752352|14737100|18803986|23122784|25157968|15163842|22046346|19228746|23220880|25435280|26432108|16397024|26487584|25221644|20658522|22493419|27697991|26243863|14967461|25238247|20124187|25694417|26628478|26920887
stop_chr17_35000001-40000000	ERBB2	AFATINIB DIMALEATE	inhibitor	ChemblInteractions	
stop_chr17_35000001-40000000	ERBB2	SORAFENIB		JAX-CKB	27992451
stop_chr17_35000001-40000000	ERBB2	ERLOTINIB		DTC|JAX-CKB|PharmGKB	27900369
stop_chr17_35000001-40000000	ERBB2	OLAPARIB		JAX-CKB|PharmGKB|FDA	
stop_chr17_35000001-40000000	ERBB2	CRIZOTINIB		JAX-CKB|CGI	27595477|26432108

stop_chr17_35000001-40000000	ERBB2	COPANLISIB		JAX-CKB	24170767
stop_chr17_35000001-40000000	ERBB2	TIVOZANIB		JAX-CKB	25995436
stop_chr17_35000001-40000000	ERBB2	IRINOTECAN		CIViC	25601188
stop_chr17_35000001-40000000	ERBB2	DACOMITINIB	inhibitor	TALC|MyCancerGenome|TdgClinicalTrial|JAX-CKB|ChemblInteractions|CGI|CIViC|GuideToPharmacology|MyCancerGenomeClinicalTrial|TTD	22135232|18606718|25899785|22761403|28363995
stop_chr17_35000001-40000000	ERBB2	DOXORUBICIN		JAX-CKB	
stop_chr17_35000001-40000000	ERBB2	FLOXURIDINE		NCI	17177846
stop_chr17_35000001-40000000	ERBB2	VINORELBINE		JAX-CKB	27992451|24742739
stop_chr17_35000001-40000000	ERBB2	TIPIRACIL		FDA	
stop_chr17_35000001-40000000	ERBB2	SIROLIMUS		CIViC	19122144
stop_chr17_35000001-40000000	ERBB2	OXALIPLATIN		JAX-CKB	28784859|26432108|24300914
stop_chr17_35000001-40000000	ERBB2	ACALABRUTINIB	inhibitor	GuideToPharmacology	
stop_chr17_35000001-40000000	ERBB2	ROMIDEPSIN		NCI	11929951
stop_chr17_35000001-40000000	ERBB2	PACLITAXEL		JAX-CKB|CIViC	24886365|26469692|27078022|24868024|27556950|26432108|29584549
stop_chr17_35000001-40000000	ERBB2	FULVESTRANT		CGI|PharmGKB|FDA	
stop_chr17_35000001-40000000	ERBB2	IXABEPILONE		PharmGKB|FDA	
stop_chr17_35000001-40000000	ERBB2	PONATINIB		JAX-CKB	26270481
stop_chr17_35000001-40000000	ERBB2	ABEMACICLIB		CGI|PharmGKB|FDA	
stop_chr17_35000001-40000000	SLFN11	NIRAPARIB		CIViC	25779942
stop_chr17_35000001-40000000	SLFN11	TEMOZOLOMIDE		CIViC	25779942|27440269
stop_chr17_35000001-40000000	SLFN11	TALAZOPARIB		CIViC	27440269
stop_chr17_35000001-40000000	CCL3	INFLIXIMAB		NCI	16219708
stop_chr19_1-5000000	TBXA2R	MORPHINE		NCI	3435201
stop_chr19_1-5000000	TBXA2R	ILOPROST	agonist	GuideToPharmacology	
stop_chr19_1-5000000	TBXA2R	FUROSEMIDE		NCI	7589163
stop_chr19_1-5000000	TBXA2R	VINBLASTINE		NCI	8632653
stop_chr19_1-5000000	TBXA2R	DINOPROSTONE	agonist	GuideToPharmacology	
stop_chr19_1-5000000	TBXA2R	CYCLOSPORINE		NCI	1385733
stop_chr19_1-5000000	TBXA2R	ASPIRIN		PharmGKB	15898979|23688183|17245331
stop_chr19_1-5000000	TBXA2R	ALPROSTADIL		TTD	
stop_chr19_1-5000000	GRIN3B	FELBAMATE	antagonist	ChemblInteractions	
stop_chr19_1-5000000	GRIN3B	KETAMINE HYDROCHLORIDE		ChemblInteractions	
stop_chr19_1-5000000	GRIN3B	ESKETAMINE		ChemblInteractions	
stop_chr19_1-5000000	GRIN3B	AMANTADINE HYDROCHLORIDE	antagonist	ChemblInteractions	
stop_chr19_1-5000000	GRIN3B	ORPHENADRINE HYDROCHLORIDE	antagonist	ChemblInteractions	
stop_chr19_1-5000000	GRIN3B	ACAMPROSATE CALCIUM	antagonist	ChemblInteractions	
stop_chr19_1-5000000	GRIN3B	ORPHENADRINE	antagonist	TdgClinicalTrial|TEND	17139284|17016423|8788072

stop_chr19_1-5000000	GRIN3B	ORPHENADRINE CITRATE	antagonist	ChemblInteractions	
stop_chr19_1-5000000	GRIN3B	ESKETAMINE HYDROCHLORIDE		TdgClinicalTrial|ChemblInteractions	
stop_chr19_1-5000000	GRIN3B	MEMANTINE HYDROCHLORIDE		ChemblInteractions	
stop_chr19_1-5000000	PLIN3	GALSULFASE		TdgClinicalTrial	17016423|18709516
stop_chr19_1-5000000	PLIN3	IDURSULFASE		TdgClinicalTrial|TEND	18201153|17139284|17016423|18038146|19707363
stop_chr19_1-5000000	AMH	TESTOSTERONE		NCI	11248089|11545286|1292985|14584782|11315948|7962305|7818099
stop_chr19_1-5000000	MKNK2	ERLOTINIB		DTC	
stop_chr19_1-5000000	MKNK2	GEFITINIB		DTC	
stop_chr19_1-5000000	MKNK2	SORAFENIB		DTC	
stop_chr19_1-5000000	PIP5K1C	ALCOHOL		PharmGKB	
stop_chr19_50000001-55000000	KIR2DS4	METHOTREXATE		PharmGKB	25069714
stop_chr19_50000001-55000000	KLK4	ECALLANTIDE		TTD	
stop_chr19_50000001-55000000	KLK4	BORTEZOMIB		TTD	
stop_chr19_50000001-55000000	CACNG6	BEPRIDIL HYDROCHLORIDE	blocker	ChemblInteractions	
stop_chr19_50000001-55000000	CACNG6	PREGABALIN	modulator	ChemblInteractions	
stop_chr19_50000001-55000000	CACNG6	GABAPENTIN ENACARBIL	modulator	ChemblInteractions	
stop_chr19_50000001-55000000	CACNG6	GABAPENTIN	modulator	ChemblInteractions	
stop_chr19_50000001-55000000	PRPF31	METFORMIN		PharmGKB	29650774
stop_chr19_50000001-55000000	NDUFA3	METFORMIN HYDROCHLORIDE	inhibitor	ChemblInteractions	
